# Differences in Mediterranean Diet Adherence between Cyclists and Triathletes in a Sample of Spanish Athletes

**DOI:** 10.3390/nu10101480

**Published:** 2018-10-11

**Authors:** José Joaquín Muros, Mikel Zabala

**Affiliations:** 1 Department of Didactics of Musical, Plastic and Corporal Expression, University of Granada, 18071 Granada, Spain; jjmuros@ugr.es; 2 Department of Physical Education, University of Granada, 18071 Granada, Spain

**Keywords:** Mediterranean diet, athletes, Spain

## Abstract

Adherence to the Mediterranean diet (MD) has rapidly declined in Mediterranean countries due to the increasing introduction of the Western diet. The aim of this study was to describe adherence to the MD within a sample of athletes from Spain. A second aim was to predict adherence to various components of the MD according to region, sex, and sport discipline. A cross-sectional study was conducted with a sample of 4037 (34.14 ± 9.28 years old) cyclists and triathletes (men: 90.1%). Participants self-reported their sex, date of birth, the number of years they had been practicing their sport, height, weight, sport discipline (cyclist, triathlon), and region. Mediterranean Diet Adherence Screener (MEDAS) was used to determine level of adherence to the MD. Women reported a higher MEDAS score and body mass index (BMI) (*p* ˂ 0.000) than men. Cyclists reported a lower MEDAS score (7.44, SD 2.12 vs. 7.85, SD 2.08), and older age (37.72, SD 9.67 vs. 34.54, SD 8.58) and BMI (23.74, SD 2.69) vs. 22.85, SD 2.28) than triathletes. The study showed that a large proportion of the surveyed athletic population were not meeting the MD guidelines, with particularly low consumption amongst men and cyclists. There were no regional effects. Nutritional guidelines for athletes should be individual rather than general and follow specifications identified by the present research.

## 1. Introduction

The Mediterranean diet (MD) is rich in vegetables, fruit, legumes, nuts, and cereals, with olive oil as the staple dietary fat. The typical MD includes moderate to high intake of fish, moderate intake of dairy products, and low consumption of meat products [1]. Adherence to the MD has been proven to have health benefits for adults, such as protection against cardiovascular disease [2], type 2 diabetes [3], and metabolic syndrome, and improving blood pressure, waist circumference, high-density lipoprotein cholesterol, triacylglycerol, and glucose concentration [4].

Despite these health benefits, adherence to the MD has been rapidly declining in Mediterranean countries [5] including Spain [6]. These countries are replacing the MD with a Western diet, which is rich in animal products, refined carbohydrates, and fat and lacking in consumption of fruit and vegetables.

The benefits of physical exercise for health are well recognised [7]. An increase in physical activity has a significant role in the prevention of diseases such as cardiovascular disease, obesity, diabetes mellitus, cancer, depression, Alzheimer disease, arthritis, and osteoporosis [8]. Studies have shown an association between high levels of physical activity and Mediterranean diet adherence [6,7,8,9]. Trends since the late 1990s also show adherence to be consistently higher in southern regions [10], partly due to the warmer climate and closer proximity to the Mediterranean Sea [11]. However, no study has conducted this analysis for athletes who participate specifically in cycling or triathlons. It is currently unknown if cyclists and triathletes demonstrate different dietary practices with relation to MD adherence.

The aim of this study was to describe adherence to the MD within a sample of physically active adults who regularly engage in cycling or triathlons from all regions of Spain. We hypothesised that there would likely be differences in the nutritional habits between participants who engaged in cycling and those who engaged in triathlons. Although both sports are endurance sports, triathlon is considered to be a new sport compared with cycling and motivations for practice are likely to be different. A second aim was to predict adherence to various components of the MD according to region, sex, and discipline.

## 2. Materials and Methods

### 2.1. Design

This study used a cross-sectional design with a convenience sample. The demographic characteristics and the Mediterranean Diet Adherence Screener (MEDAS) were introduced into the application Google Drive^®^ (Alphabet, Mountain View, CA, USA). The final questionnaire and instructions were sent by e-mail to the Royal Spanish Cycling Federation and to the Spanish Triathlon Federation, who forwarded them to all of their associated members. To be eligible for inclusion, participants had to be over 18 years old and have previously provided permission to their Federation to send e-mails to them. Research was conducted in 2016.

We adhered to the ethical principles of the Declaration of Helsinki for medical research. Ethical approval was granted by the Ethics Committee of the University of Granada, Spain (approval code: 883).

### 2.2. Subjects

A sample of 4037 (36.14 ± 9.28 years old) cyclists and triathletes (male: 90.1%) from across Spain (Table 1) was used for this study. There were 75,871 cyclists (male: 95%) federated in Spain during 2016 of which 2037 (male: 95.5%) satisfactorily completed the questionnaire and 27,760 triathletes (male: 82.3%) federated in Spain during 2016 of which 2000 (male: 84.5%) satisfactorily completed the questionnaire.

### 2.3. Variables

Participants were asked to self-report their sex, data of birth, years practicing their sport, marital status, height, weight, sport discipline (cyclist, triathlon), and region (North: Aragon, Asturias, Balearic Islands, Basque Country, Cantabria, Castile and Leon, Catalonia, Community of Madrid, Galicia, La Rioja, or Navarre; South: Andalusia, Canary Islands, Castile-La Mancha, Ceuta, Melilla, Extremadura, Region of Murcia, and Valencian Country). Body mass index (BMI) was calculated as weight divided by height squared (kg/m^2^). For ease of interpretation, categorical variables of sex (men and women), sport discipline (cyclists and triathlon), and region (north and south) were dichotomised. A total of 11% of the participants lived alone. Regarding marital status, 46.4% were single, 49.8% married, and 3.7% divorced.

MEDAS [12] was used to determine the level of adherence to the MD. This questionnaire consists of 14 items related to Mediterranean dietary patterns, 12 questions on food consumption frequency, and 2 questions on food intake habits, according to characteristics of the Spanish Mediterranean diet. Each question was scored 0 or 1 producing a derived score ranging from 0 to 14. One point was given for using olive oil as the principal source of fat for cooking and one for preferring white meat over red meat. One point was given for consumptions of: four or more tablespoon of olive oil per day; two or more servings of vegetables per day; three or more pieces of fruit per day; less than one serving of red meat, burgers, or sausages per day; less than one serving of butter, margarine, or cream per day; less than one carbonated and/or sugar-sweetened beverage per day; seven or more cups (100 mL) of wine per week; three or more servings of pulses per week; three or more servings of fish/seafood per week; less than two commercial pastries such as cookies or cakes per week; three or more servings of nuts per week; and two or more servings per week of boiled vegetables, pasta, rice, or other dishes with sauce, tomatoes, garlic, onion, or leeks sautéed in olive oil. The higher the score, the greater the adherence to the MD. Two MEDAS items were not included in the binary logistic regression model due to the lack of variation in responses, with more than 95% reporting using olive oil as the principal source of fat and less than 5% reporting meeting recommendations for consumption of wine. Although MEDAS was designed to evaluate elderly people, it is also suitable for assessing MD adherence in younger adults and adults [13].

### 2.4. Statistical Analysis

The mean for all quantitative variables is presented alongside the standard deviation. Normality of the data was tested using the Kolmogorov-Smirnov test with Lilliefort’s correction and homoscedasticity was assessed using the Levene test. After verifying that the variables were non-normally distributed, the data were analysed using the U Mann-Whitney test for two-group comparison. Categorical variables are presented according to their frequency distribution and associations between them were determined using the Chi-square test.

Three binary logistic regression models were used to predict the probability of observations of the 12 items of the MEDAS according to sex, sport discipline, and region: Model 1: region (north and south) was entered as the predictor variable, representing a basic model adjusted for years practicing the relevant sport, BMI, sex, and sport discipline; Model 2: sport discipline was entered as the predictor variable, which was a basic model adjusted for years practicing the relevant sport, BMI, sex and region; and Model 3: sex was entered as the predictor variable, which was a basic model adjusted for years practicing the relevant sport, BMI, sport discipline, and region. Model fit was assessed using Nagelkerke *R*^2^. The model demonstrated acceptable fit to data: Model 1 *R*^2^: 0.269, Model 2 *R*^2^: 0.336, and Model 3 *R*^2^: 0.310. Data were analysed using the IBM-SPSS version 22.0 statistical programme for Windows (Armonk, NY, USA: IBM Corp.). The level of significance was set at 0.05.

## 3. Results

Data for MEDAS overall score, age, BMI, and time practicing the relevant sport for all study participants according to region, sex, and sport discipline are shown in Table 1. There were no significant differences according to region (north vs. south) in terms of MEDAS overall score, sex, and sport discipline. Northern participants were significantly older (*p* ˂ 0.01), with lower BMI (*p* ˂ 0.05), and reported more time practicing their relevant sport (*p* ˂ 0.001). There were significant differences according to sex in relation to MEDAS overall score, age, BMI, and time practicing the relevant sport (*p* ˂ 0.000). Women reported a higher MEDAS score and BMI, and lower age and time practicing their relevant sports. According to discipline, cyclists reported a lower MEDAS score and higher age, BMI, and time practicing their sport (*p* ˂ 0.000).

Binary logistic regression analysis was conducted in order to predict adherence to various components of the MD as described by the different items on the MD questionnaire (Table 2). Participants who lived in the north were less likely than those living in the south to report meeting guidelines for the weekly consumption of olive oil (odds ratio (OR) = 0.75, 95% confidence interval (CI) = 0.64, 0.87); red meat, burgers, or sausages (OR = 0.75, 95% CI = 0.64, 0.87); nuts (OR = 0.86, 95% CI = 0.76, 0.99); and boiled vegetables, pasta, rice, or other dishes with tomato sauce, garlic, onion, or leeks sautéed in olive oil (OR = 0.85, 95% CI = 0.73, 0.99). Participants living in the north were more likely to meet recommendations for consumption of butter, margarine, or cream (OR = 1.48, 95% CI = 1.25, 1.75) and carbonated and/or sugar-sweetened beverages (OR = 1.44, 95% CI = 1.25, 1.65). They were also more likely to report preferring white meat over red meat (OR = 0.66, 95% CI = 0.55, 0.78). According to sport discipline, cyclists were less likely than triathletes to meet recommendations for consumption of fruit (OR = 0.74, 95% CI = 0.63, 0.88), carbonated and/or sugar-sweetened beverages (OR = 0.74, 95% CI = 0.63, 0.88), and nuts (OR = 0.67, 95% CI = 0.57, 0.79). Cyclists were more likely than triathletes to meet recommendations for boiled vegetables, pasta, rice, or other dishes with tomato sauce, garlic, onion, or leeks sautéed in olive oil (OR = 1.23, 95% CI = 1.01, 1.49). Model 3 considered sex as a predictor. Women were less likely than men to meet recommendations for consumption of pulses (OR = 0.68, 95% CI = 0.51, 0.90), nuts (OR = 0.78, 95% CI = 0.61, 0.99), and boiled vegetables, pasta, rice, or other dishes with tomato sauce, garlic, onion, or leeks sautéed in olive oil (OR = 0.53, 95% CI = 0.42, 0.69). Women were more likely to meet recommendation for consumption of vegetables per day (OR = 2.22, 95% CI = 1.72, 2.85) and red meat, burgers, or sausages per day (OR = 1.44, 95% CI = 1.11, 1.86).

## 4. Discussion

One of the main findings highlighted in this study was that a large proportion of the measured population did not meet MD guidelines. We also examined different individual aspects of the MD guidelines according to sex, sport discipline, and region.

The overall mean MEDAS score for the present sample of Spanish cyclists and triathletes was 7.64, higher than 6.34, which was reported in a representative sample of non-institutionalised Spanish adults aged 18 years old or older [6]. Although the overall MEDAS score was higher than that previously reported in a Spanish sample, it is still short of describing strict accordance to the MD (≥9 points) [14]. Abandonment of MD habits have been previously reported in Mediterranean countries such as Spain [6], Greece [15], and Italy [16]. These countries are slowly replacing their traditional patterns of eating with Western dietary patterns rich in animal products, refined carbohydrates, and fat, and low consumption of fruit and vegetables. Levels of physical activity could explain differences in the overall MEDAS score of the present sample and that reported previously in another sample of non-institutionalised Spanish adults. Previous studies showed associations between high MD scores and high levels of daily physical activity, including in a sample of European adults [17] and a Spanish sample [18]. Another study found that cycling was associated with higher values of adherence to a MD, irrespective of training volume [19].

In the present study, there were no significant differences according to region (north vs. south) in terms of overall MEDAS score. The present findings contradict those previously reported by Abellán Alemán et al. [20], which showed that South-eastern Spain had the lowest score for adherence to the MD due to low consumption of fish and plant products. A study by Bach-Faig et al. [10] reported MD adherence over 20 years to be significantly higher in some southern Mediterranean regions of Spain, such as Andalusia. Teenagers from the southern region of Italy also showed the highest regional adherence for their country [21]. The different samples examined in these studies could explain the differences reported, as the present study was conducted with a sample of athletes and a physically active lifestyle was previously associated with a greater adherence to the MD independent of region [18,22]. Other potential confounders, such as socio-economic and educational status, should also be considered, as previous studies demonstrated that individuals with low socioeconomic and educational levels report low consumption of fish, fruit, and fresh vegetables [23,24]. In our study, those participants who were living in the northern regions were less likely than those living in the south to report meeting guidelines for the weekly consumption of olive oil. Although more than 95% of participants in both regions used olive oil as the principal source of fat for cooking, 78.5% participants who were living in the south consumed four or more tablespoons of olive oil per day in comparison to 74.1% of the participants who lived in the north. Also, participants who lived in the south consumed more boiled vegetables, pasta, rice, or other dishes with tomato sauce, garlic, onion, or leeks sautéed in olive oil. This can be explained by the finding that southern participants used more olive oil as oil for cooking. Participants who lived in the north were less likely than those living in the south to report meeting guidelines for red meat consumption. This corroborates previous results that showed that people living in the lower northern areas of Spain consumed more red meat and dairy products [20]. However, participants who lived in the south were also less likely to meet the recommendation for consumption of carbonated and or sugar-sweetened beverages. A number of factors could explain these findings. It is possible than the warmer climate contributes to a greater consumption of these beverages.

Women received a higher MEDAS score than men. A systematic review within Greek and Cypriot populations did not find statistically significant differences according to sex [25]. Contrasting results were found in a sample from Catalonia [26] and in a representative sample of Spanish adults in which women had lower adherence to the MD than men. This difference was mostly due to a lower consumption of wine amongst women with differences disappearing when wine was excluded from MD calculations [6]. Differences in the present research could also be related to the nutritional habits and knowledge of the included athletes. A comparison study with collegiate athletes previously showed that male athletes consume fast food or restaurant meals more frequently and have higher and more frequent weekly alcohol intake during the competitive season than women [27]. Female Australian elite athletes also reported a better diet quality than male athletes in addition to better nutrition knowledge [28]. A previous systematic review also reported that athletes’ knowledge was equal to or better than non-athletes and that this knowledge was greater in women than in men [29]. Regarding specific components of the MD, women were more likely than men to meet the recommendations for daily consumption of vegetables (59.1% vs. 36.1%) and red meat (34.9% vs. 23.2%). Similar results were found by de Boer et al. [30] who concluded that differences could be driven by greater health consideration and awareness of climate change amongst women. Recommendations for consumption of pulses and fish should be given special consideration as the percentage of athletes following this MD recommendation in the present study was very small (less than 30% for pulses and less than 40% for fish), regardless of sex. Our results correspond with results reported for the general population of Spain, which suggest 22% meet the recommendation for pulses and only 30.2% consume three to four portions of fish/seafood per week [20].

With regards to discipline, triathletes had a higher MEDAS scored than cyclists. No previous studies reported adherence to the MD in a sample of triathletes and only two studies reported adherence to the MD in cyclists. One of these reported higher adherence to the MD amongst cyclists than amongst inactive adults [19]. Similar results were found in the second study, in which young male cyclists reported higher adherence to the MD than a matched group of non-cyclists [31]. In the present study, triathletes were more likely to meet the recommendation for consumption of fruit, nuts, and carbonated and or sugar-sweetened beverages than cyclists. This could be related to motives for participating in different endurance sports. Many cyclists were motivated by competition, whereas triathletes tended to be motivated by fitness, health benefits, and weight loss [32].

Regarding the recommendation for wine consumption, our results found that less than 5% of all surveyed athletes followed this guideline independent of sex, age, region, or discipline. Several factors could explain these findings. Firstly, alcohol is included on the list of prohibited substances and methods for athletes, meaning that they only occasionally consume alcohol [33]. The proportion of athletes that never consumed alcohol was higher than in a regular population and teetotal individuals would not be encouraged to commence drinking [34]. Despite the benefits of red wine on heart and kidney protection from ischemia-reperfusion injury [35], the recommendation concerning wine within this type of population should be optional and always with meals.

Two main recommendations emerge from the present findings. Firstly, although nutritional policies in Mediterranean countries have focused on the preservation of MD, nutritional intervention evidently requires comprehensive and specific messages about the different components of this diet. Specific recommendations should be elaborated for endurance sport athletes and specific recommendations for other sports should also be studied. 

Limitations of the present study include its cross-sectional design, which precludes drawing conclusions on the direction of associations and inhibits the investigation of causal relationships. Adherence to a Mediterranean diet was measured using a validated questionnaire. The questionnaire has inherent limitations of precision due to reliance on self-reported data and the choice of research method, which precludes independent verification. However, the risk of error was minimized by ensuring anonymity of responses. The use of self-reporting to assess a number of variables increases the possibility of measurement error. However, MEDAS has previously demonstrated high validity and reliability in similar populations. The questionnaire was validated alongside the established food frequency questionnaire (FFQ), producing an average MEDAS score estimate of 105% of the FFQ score estimate suggesting that it is a valid instrument for rapid estimation of adherence to the MD. Weight and height were self-reported as opposed to directly measured due to time, financial resources, and labor constraints. Although this method is less accurate than direct measurement, it has demonstrated good agreement and validity in healthy weight populations. It was not possible to evaluate the socioeconomic status (SES) of individuals in this study. Future studies should measure SES where possible.

## 5. Conclusions

To the best of our knowledge, this is one of few investigations that describe adherence to a Mediterranean diet in a sample of athletes, and this is the first to analyse differences between cyclists and triathletes in a sample of Spanish athletes. This study is also the first to describe different nutritional patterns according to region, sex, and sport discipline in a sample of athletes. Future interventions should focus on an increase in vegetables and a decrease in red meat and hamburgers or sausages as a source of protein, especially in men. Another important focus of nutritional interventions with athletes should be an increase in pulses and fish/seafood independent of sex, region, or discipline, an increase in the consumption of fruit, and decreased consumption of carbonated sugar-sweetened drinks amongst cyclists. The study showed that a large proportion of the surveyed athletic population were not meeting MD guidelines, with particularly low consumption amongst men and cyclists. The findings have important implications for the design of nutritional interventions for athletes across Spain. It is essential to provide nutritional guidelines to athletes that consider the present findings rather than just promoting the MD in general.

## Figures and Tables

**Table 1:** Characteristics of study sample.

Characteristic	MEDAS Points (SD)	Male *N* (%)	Cyclists *N* (%)	Age Years (SD)	BMI kg/m^2^ (SD)	Time Practicing Years (SD)
Overall (*n* = 4037)	7.64 (2.11)	3636 (90.07)	2037 (50.46)	36.14 (9.28)	23.30 (2.54)	9.31 (9.18)
North (*n* = 2340)	7.63 (2.09)	2108 (90.08)	1156 (49.40)	36.53 (9.63)	23.22 (2.53)	9.75 (9.61)
Aragon (*n* = 199)	7.59 (1.83)	184 (92.46)	125 (62.81)	37.13 (9.30)	23.25 (2.41)	9.86 (8.71)
Asturias (*n* = 160)	7.43 (1.88)	150 (93.75)	117 (73.13)	34.89 (10.10)	23.45 (2.74)	11.44 (9.64)
Balearic Islands (*n* = 112)	7.39 (2.00)	104 (92.86)	50 (44.64)	36.45 (9.14)	23.45 (2.22)	8.08 (8.53)
Basque Country (*n* = 265)	7.83 (1.90)	241 (90.94)	119 (44.91)	36.31 (9.29)	23.24 (2.41)	10.55 (9.80)
Cantabria (*n* = 135)	7.22 (2.09)	130 (96.30)	133 (98.52)	37.71 (8.33)	23.99 (2.33)	11.71 (10.31)
Castile and Leon (*n* = 268)	7.80 (2.24)	248 (92.54)	173 (64.55)	36.65 (10.32)	23.24 (2.52)	11.91 (10.58)
Catalonia (*n* = 476)	7.76 (2.03)	393 (82.56)	160 (33.61)	37.06 (9.84)	23.10 (2.41)	8.53 (9.57)
Community of Madrid (*n* = 452)	7.60 (2.18)	404 (89.38)	166 (36.73)	37.17 (9.51)	23.14 (2.81)	8.84 (9.39)
Galicia (*n* = 203)	7.38 (2.24)	185 (91.13)	86 (42.36)	34.08 (9.19)	22.93 (2.56)	8.74 (8.61)
La Rioja (*n* = 13)	7.62 (1.61)	11 (84.62)	0 (0.00)	36.08 (6.56)	23.02 (2.88)	3.54 (3.97)
Navarre (*n* = 61)	7.97 (1.91)	58 (95.08)	27 (44.26)	36.18 (11.10	22.79 (1.97)	11.46 (10.07)
South (*n* = 1693)	7.66 (2.12)	1528 (90.25)	881 (52.04)	35.61 (8.76)	23.40 (2.55)	8.70 (8.51)
Andalusia (*n* = 971)	7.61 (2.18)	906 (93.31)	680 (70.03)	36.39 (8.83)	23.61 (2.66)	9.76 (8.86)
Canary Islands (*n* = 137)	7.30 (2.11)	125 (91.24)	55 (40.15)	35.44 (8.34)	23.46 (2.69)	8.24 (7.76)
Castile-La Mancha (*n* = 58)	7.90 (1.92)	50 (86.21)	5 (8.62)	31.98 (7.63)	23.04 (2.61)	4.34 (4.09)
Ceuta (*n* = 2)	9.00 (0.00)	2 (100.0)	0 (0.00)	41.50 (6.36)	22.49 (0.89)	15.50 (10.61)
Melilla (*n* = 11)	6.82 (1.94)	10 (90.91)	8 (72.73)	37.00 (6.45)	25.47 (3.08)	6.82 (7.47)
Extremadura (*n* = 132)	7.33 (2.15)	120 (90.91)	96 (72.73)	36.18 (9.98)	23.42 (2.48)	10.55 (9.06)
Region of Murcia (*n* = 105)	7.76 (1.94)	93 (88.57)	34 (32.38)	33.85 (8.40)	22.93 (2.24)	8.46 (8.86)
Valencian Country (*n* = 277)	8.12 (1.95)	222 (80.14)	3 (1.08)	33.98 (8.01)	22.81 (2.01)	5.35 (6.50)
*p*-value	0.466	0.736	0.088	0.001	0.042	0.008
Men (*n* = 3636)	7.59		1946 (53.52)	36.60 (9.15)	23.54 (2.46)	9.66 (9.31)
Women (*n* = 401)	8.15		91 (22.69)	31.96 (9.40)	21.08 (2.16)	6.13 (7.17)
*p*-value	0.000		0.000	0.000	0.000	0.000
Cyclists (*n* = 2037)	7.44 (2.12)	1946 (95.53)		37.72 (9.67)	23.74 (2.69)	12.98 (10.03)
Triathletes (*n* = 2000)	7.85 (2.08)	1690 (84.50)		34.54 (8.58)	22.85 (2.28)	5.56 (6.31)
*p*-value	0.000	0.000		0.000	0.000	0.000

MEDAS: Mediterranean Diet Adherence Screener; BMI: Body mass index.

**Table 2:** Binary logistic regression for the different aspects of the Mediterranean diet.

Variables	Model 1			Model 2			Model 3		
	OR	CI (95%)	*p* Value	OR	CI (95%)	*p* Value	OR	CI (95%)	*p* Value
Do you consume ≥4 tablespoon of olive oil per day?	0.75	0.64–0.87	0.000	1.20	0.99–1.45	0.061	1.14	0.85–1.53	0.374
Do you consume ≥2 servings of vegetables per day?	0.92	0.80–1.05	0.215	0.91	0.76–1.08	0.283	2.22	1.72–2.85	0.000
Do you consume ≥3 pieces of fruit per day?	1.07	0.94–1.23	0.300	0.74	0.63–0.88	0.000	0.91	0.70–1.17	0.458
Do you consume ˂1 serving of red meat, hamburger, or sausages per day?	0.75	0.64–0.87	0.000	1.06	0.88–1.28	0.532	1.44	1.11–1.86	0.006
Do you consume ˂1 serving of butter, margarine, or cream per day?	1.48	1.25–1.75	0.000	1.06	0.87–1.31	0.558	0.99	0.72–1.38	0.963
Do you consume ˂1 carbonated and/or sugar-sweetened beverages per day?	1.44	1.25–1.65	0.000	0.74	0.63–0.88	0.001	1.31	0.99–1.73	0.059
Do you consume ≥3 serving of pulses per week?	1.06	0.92–1.23	0.429	1.11	0.93–1.33	0.252	0.68	0.51–0.90	0.008
Do you consume ≥3 serving of fish/seafood per week?	1.12	0.97–1.29	0.125	0.88	0.74–1.05	0.157	1.07	0.83–1.37	0.597
Do you consume ˂2 commercial pastry such as cookies or cake per week?	0.93	0.81–1.06	0.280	1.06	0.90–1.25	0.500	1.19	0.93–1.54	0.174
Do you consume ≥3 serving of nuts per week?	0.86	0.76–0.99	0.030	0.67	0.57–0.79	0.000	0.78	0.61–0.99	0.047
Do you prefer to eat chicken, turkey, or rabbit instead of beef, pork, hamburgers, or sausages?	0.66	0.55–0.78	0.000	0.92	0.75–1.14	0.453	1.36	0.97–1.90	0.071
Do you consume ≥2 times per week boiled vegetables, pasta, rice, or other dishes with a sauce tomato, garlic, onion, or leeks sautéed in olive oil?	0.85	0.73–0.99	0.046	1.23	1.01–1.49	0.037	0.53	0.42–0.69	0.000

OR: Odds Ratio; CI: Confidence Interval.
